# Maurice H. Collis

**Published:** 1869-05

**Authors:** 


Maurice H. Collis, Surgeon to the Meath Hospital, Dublin, the author of a valuable work upon Cancer and Tumors, died a few weeks since from pyæmic poisoning consequent upon a slight scratch received by him whilst removing an upper jaw in the operating theatre of his hospital. Mr. Collis was in his forty-fifth year.

